# Sexual Function, Mental Well-being and Quality of Life among Kurdish Circumcised Women in Iran

**Published:** 2017-09

**Authors:** Farzaneh DANESHKHAH, Hamid ALLAHVERDIPOUR, Leila JAHANGIRI, Tatiana ANDREEVA

**Affiliations:** 1. Dept. of Health Education & Promotion, Tabriz University of Medical Sciences, Tabriz, Iran; 2. Clinical Psychiatry Research Center, Tabriz University of Medical Sciences, Tabriz, Iran; 3. School of Public Health, National University of Kyiv-Mohyla Academy, Kiev, Ukraine

**Keywords:** Female circumcision, Circumcision, Sexual function, Women’s health

## Abstract

**Background:**

Female genital mutilation is an intentional inhumane procedure that threatens girls and women’s health. It is especially widespread in developing countries due to cultural, traditional and religious preferences. The aim of the current study was to investigate how circumcision affects women’s sexual function.

**Methods:**

This cross-sectional study was conducted in the urban and rural area of Piranshahr County, Iran, in 2015 among convenience samples of 200 women, 15–49 yr old, who were applying to health care centers for receiving routine health care services. Data collection was conducted with the use of a self-administered written questionnaire to assess female sexual function, mental well-being, and quality of life.

**Results:**

Significant differences were found between circumcised and non-circumcised women in total score of female sexual function index (FSFI) in domains of desire, arousal, vaginal moisture, orgasm, satisfaction, and pain [(*P*<0.001), MD(95%CI)=5.64(3.64 to 7.64)] and based on Hotelling’s T-square, significant differences were found in dimensions of quality of life and FSFI.

**Conclusion:**

The revealed sexual dysfunction among mutilated women gives ground to require that public health systems take actions aimed at implementing special sexual education program to improve sexual functions of mutilated women and changing beliefs and social norms in the community level.

## Introduction

Many girls born in countries of Africa and Middle East undergo inhuman procedure known as Female Genital Mutilation/Cutting (FGM/C). “FGM/C refers to all the procedures that intentionally alter female genitalia including their partial or total removal for non-medical purposes” (1).

About 100–140 million girls and women have undergone FGM and are suffering FGM consequences globally. FGM currently occurs in 28 African countries affecting 67.7 million girls and women who are currently 15–49 yr old and more than three million girls have been estimated to be at risk for FGM annually (1, 2). In addition to African countries, this phenomenon is seen in the East Asian countries such as Indonesia, Malaysia, and India as well as in the Middle East and among migrants from these areas to Europe, the USA, Australia and other countries where migrants carry along their own traditions (3–6). The described practices need to be terminated based on principles stated in the Universal Declaration of Human Rights article five saying that “no one shall be subjected to torture or cruel, inhuman or degrading treatment” (7).

History and the rationale why FGM is practiced in certain nations, religions, and cultures are not sufficiently understood except for the fact that female genital mutilation was known since ancient Egypt (8). Current observations show that FGM is common in some Islamic countries (9). The prerequisites for female genital mutilation are related to a mix of cultural, customary, traditional, religious and social factors within families and communities, perceived as ancestral socio-cultural roots (1). The rationale for performing FGM/C includes preservation of ethnic and gender identity, maintenance of ‘cleanliness’ and assurance of women’s virginity, along with the control of women’s sexuality. FGM/C is also considered as a rite of passage from childhood to adulthood (3, 10, 11).

FGM harms girls and women in many ways (1, 10, 12). In others words, FGM is a risk factor for several adverse health conditions among women, and all types of FGM procedures have immediate and long-term health consequences (13). Immediate complication of FGM includes severe pain, shock, hemorrhage, and infection that can lead to septicemia and acute urine retention, psychological consequences, as well as open sores in the genital region (3, 9, 11). In a long run, a range of traumatic consequences can arise including genital ulcers, keloid scars, fibrosis, and chronic vulvar pain; inflammatory consequences include recurrent bladder infections; menstrual difficulties which might manifest as painful and prolonged periods; and psychosexual consequences including dyspareunia and sexual dysfunction (3, 14). With three types of FGM being distinguished (1), most severe complications arise with types II and III procedures and especially type III that include repeated urinary tract and pelvic infections, urethra damage, infertility, an increased risk of childbirth complications, and natal deaths (15, 16). Additionally, various sexual dysfunctions as a component of FGM consequences affect women’s emotional and sexual well-being (16–19).

The above-mentioned evidence of FGM devastating impact became the ground for the UN declaration on the elimination of FGM and the WHO commitment to provide a “Global strategy to stop health care providers from performing female genital mutilation” (1), which calls all health professionals to act to prevent this harmful and violent practice which is inhuman and threatening to women’s human rights.

FGM is known as a traumatic procedure that can demolish and impair sexual functions of mutilated women including altered desire/libido, pain/discomfort, and diminished arousal resulting in inhibited orgasm(3, 14) while the British Medical Association concluded that little is documented with regard to the psychosexual and psychological consequences of FGM (20). Additionally, not much has yet been documented with regard to psychosexual outcomes of FGM specific for different nations and regions. In Iran, with a great variety of national, racial, and religious groups, FGM predominantly occurs in the west and south of Iran where most of the people are Sunni (the largest branch of Islam) or belong to Kurdish nation.

Based on the above mentioned background, the aims of the current study were to obtain information about the consequences of female circumcision on sexual function of circumcised women. We also aimed to acquire explorative information regarding the status of FGM, its prevalence, predominant method/type of female circumcision, and attitude to this practice among the Kurdish women in Iran.

## Materials and Methods

### Participants and Procedures

This cross-sectional study was performed in Piranshahr County, Iran, near the border of Iraq where a part of Kurdish people is living there. Participants (n=200) were sexually active married women of child-bearing age 15–49. Convenience sampling was conducted through two urban and five rural health centers; all the women who applied to health care centers for routine health care services during the data collection period and agreed to participate were recruited. Data were collected during Feb and Mar 2015 by means of in-person home-based interviews guided with a structured written questionnaire and clinical examination by a midwife. In case of needing to clinical examination, participants were examined by a midwife in the clinic. All participants were covered with governmental health care services. The age of women ranged between 15 and 49 yr; 9.5% of women were younger than 20 yr, 42% were 21–30 yr old, 37% were 31–40 yr old, and 11.5% were older than 41 yr. Tabriz University of Medical Sciences’ Ethics Committee approved the study, and informed consent was obtained from all study participants before the interview and clinical examination.

### Measures

#### Socio-demographic variables

Socio-demographic variables included age, duration of marriage, employment status (employed, housewife), education (illiterate, primary, secondary education, high school and higher), number of deliveries, place of living (rural or urban area) and parents’ education level.

### History of FGM

This was defined as any partial or total removal of the external female genitalia during the childhood age. In addition, for determining the type of FGM, participating women were asked about which parts of their genital organs were removed, cutting tool, circumcised, and age they have undergone FGM. In case of needing to diagnose the type of FGM, participants were under examination by a midwife. Additionally, viewpoints of circumcised women about why they had been under FGM were obtained by a brief semi-structured interview.

### Female sexual function index (FSFI)

The FSFI is a brief, multidimensional, validated tool for assessment of sexual function and consists of nineteen items for six domains of libido, arousal, lubrication, orgasm, satisfaction, and pain (e.g. “Over the past 4 wk, how often did you feel sexual desire or interest? Almost always or always; Most of the time (more than half of the time); Sometimes (about half the time); On a few occasions (less than half of the time); Almost never or never”). Each item has six possible answers that describe the status of sexual function during the last four weeks (14, 21). Responses to each question related to the previous month were recorded and scored either from 0 (no sexual activity) or 1 (suggestive of dysfunction) to 5 (suggestive of normal sexual activity). The reliability coefficient for the scale was 0.95. Higher scores on the scale indicated normal sexual activity.

### Quality of Life (QoL)

A 26-item rating scale was used to gauge women’s perception of their quality of life (QoL) in four domains of Physical health, Psychological, Social relationship, and Environment (e.g. ‘‘How would you rate your quality of life? Very poor; Poor; Neither poor nor good; Good; Very Good’’). A 5-point Likert-type scaling (1=strongly disagree, 5=strongly agree and 1=never, 5=completely) was used (22). The reliability coefficient for the scale was 0.94. Higher scores on the scale indicated better QoL.

### Mental wellbeing

The validated 28-item Farsi version of the General Health Questionnaire (GHQ-28) (23)was used to measure the wellbeing of women. The GHQ refers to subjective symptoms of psychological distress, somatic manifestations often associated with anxiety and depression, relationship difficulties, and perception of social, family, and professional roles (24). The GHQ-28 is composed of four subscales used to measure somatization, anxiety, social dysfunction, and depression. Both subscales and summated total scores were used (24, 25). All items had ordinal four-point Likert-type scales (0-1-2-3). A higher score on the GHQ-28 represents poorer mental health status. The reliability of coefficients of reliability of the subscales varies around 0.87 and the internal consistency of the total scale is 0.90.

### Statistical analysis

Information obtained from the two groups was analyzed using SPSS version 16 for Microsoft Windows (Chicago, IL, USA). The statistics used to describe the groups included mean, standard deviation, frequencies (number of cases) and percentages where appropriate. Comparison of quantitative variables between circumcised and non-circumcised women was conducted using Student’s t-test for independent samples. To compare categorical data, Chi-square test of independence was performed. Exact Fisher’s test was used instead when the expected frequencies in some subgroups were less than 5. The Hotelling’s T-square method was applied for simultaneous comparisons of dimensions between FGM and non-FGM women and finally, bivariate analysis was performed to ascertain the association between the quality of life and general health with sexual function trait. The correlations between low quality of life as well as low level of general health and low sexual function trait were assessed using Pearson correlation. In all analyses, *P*<0.05 was considered statistically significant.

## Results

In our sample, the total rate of FGM was 70% (n=140). With 53.6% (n=75) among women of older than 31 yr age and 8.6% (n=12) in the less than 20 yr old group. Among the rural women, 93 (66.4%) were circumcised versus 13 (22.7%) among the women in the urban area. Among the uncircumcised women, 17 (28.5%) were employed while it was true about 19 (13.3%) of circumcised women (Table 1).

**Table 1:** Demographic characteristics of noncircumcised and circumcised women

**Variables**		**Non FGM (n=60) n(%)**	**FGM (N=140) n(%)**	***P*-Value**
**Age (yr)**	Less than 20	7(11.7)	12(8.6)	0.048
	20–30	31(51.7)	53(37.9)	
	31–49	22(36.7)	75(53.6)	
**Occupation**	Housewife	43 (71.7)	121(86.4)	0.013
	Employed	(28.3)17	19(13.6)	
**Education**	Illiterate	1(1.7)	34(24.3)	<0.000
	Primary	7(11.7)	48(34.3)	
	secondary	13(21.6)	23(16.4)	
	high school	21(35.0)	21(15.0)	
	More	18(30.0)	14(10.0)	
**Mother’s education**	illiterate	37(61.7)	125(89.3)	<0.001
	primary	6(10.0)	10(7.1)	
	secondary	4(6.7)	5(3.6)	
	high school	9(15.0)	0(0.0)	
	more	4(6.7)	0(0.0)	
**Father’s education**	illiterate	45.0(27)	100(71.5)	<0.000
	primary	8(13.3)	29(20.7)	
	secondary	5(8.4)	2(1.4)	
	high school	12(20.0)	8(5.7)	
	more	8(13.3)	1(0.7)	
**Housing**	Owned	45(75.0)	123(87.9)	0.023
	Rented	15(25.0)	17(12.1)	
**Residence**	Urban	47(78.3)	47(33.6)	<0.000
	Rural	13(21.7)	93(66.4)	
**Income**	Low	19(31.7)	28(20.0)	0.115
	Middle	39(65.0)	100(71.4)	
	High	2(3.3)	12(8.6)	
		Mean(SD)	Mean(SD)	
**Duration of marriage**		6.66(0.85)	10.52(0.62)	
**Number of children**		1.18(0.14)	1.88 (0.09)	

In addition, 125 (89.3%) of participants with history of FGM reported that their mothers were illiterate versus 37 (61.7%) among uncircumcised women and participants who had literate mother were not circumcised. Table 2 shows the average scores for the participating women’s sexual function: a significant difference was found (*P*<0.001) between circumcised (mean=18.25, SD=6.32) and non-circumcised (mean=23.90, SD=7.12) women. Additionally, significant differences were found between two groups in all the domains of sexual function scale including desire, arousal, vaginal moisture, orgasm, satisfaction and pain with circumcised women having lower sexual function scores. However, no significant differences between the two groups were found in the quality of life score: (mean=52.73, SD=12.59) among circumcised women and (mean=53.39, SD=12.13) among those non-circumcised. Additionally, average scores for all the domains of quality of life are brought in Table 2. The calculated scores for general health status did not reveal significant differences between the two groups of participants (*P*-value: 0.935).

**Table 2:** Comparison of female sexual function index, quality of life, and mental wellbeing between two groups of circumcised and non-circumcised women

**Variable**		**Mean (SD)**		MD^**^ (95%CI)	**P-Value**
		FGM^*^ (n=140)	**Non FGM (n=60)**		
**FSFI**	Desire	2.67(1.07)	3.67(1.43)	0.99(0.63 to 1.35)	<0.001
	Arousal	2.85(1.30)	3.95(1.46)	1.09(0.67 to 1.50)	<0.001
	Vaginal moisture	3.02(1.18)	4.01(1.37)	0.98(0.61 to 1.36)	<0.001
	Orgasm	2.32(1.19)	3.64(1.32)	1.32(0.94 to 1.69)	<0.001
	Satisfaction	3.60(1.54)	4.34(1.45)	0.74(0.28 to 1.20)	0.002
	Pain	3.78(1.56)	4.28(1.14)	0.50(0.06 to 0.94)	0.025
	Total	18.25(6.32)	23.90(7.12)	5.64(3.64 to 7.64)	<0.001
**WHOQOL-BREF**	General Health	63.12(20.57)	64.16(17.14)	1.04(−4.92 to 7.01)	0.731
	Physical Health	62.88(15.96)	61.48(15.22)	−1.39(−6.18 to 3.39)	0.567
	Psychological	56.96(16.52)	59.86(14.39)	2.89(−1.94 to 7.74)	0.240
	Social Relationship	21.45(5.65)	22.60(6.44)	1.15(−0.64 to 2.94)	0.207
	Environment	59.24(14.50)	58.85(15.06)	−0.38(−4.85 to 4.07)	0.864
	Total	52.73(12.59)	53.39(12.13)	0.66(−3.12 to 4.45)	0.731
**GHQ-28**	Somatic symptoms	5.99(3.71)	6.58(3.78)	0.59(−0.54 to 1.72)	0.307
	Anxiety and insomnia	5.98(4.17)	6.20(4.29)	0.21(−1.06 to 1.49)	0.742
	Social dysfunction	7.30(1.62)	7.21(1.15)	−0.09(−0.54 to 0.36)	0.696
	Severe depression	4.87(4.70)	4.30(4.27)	−0.57(−1.97 to 0.81)	0.414
	Total	24.16(10.90)	24.30(10.43)	0.13(−3.14 to3.41)	0.935

* FGM - Female Genital Mutilation

** MD - mean difference

CI : Confidence interval

### *P*-Value based on independent samples t-test

Assessment of sexual behavior in two groups (Table 3) showed that 68 (48.8%) of circumcised women had low desire for having a sexual relationship while only 16 (26.7%) were similarly characterized among the non-circumcised. Regarding women’s demand or offer for sexual contact, 15 (25%) of non-circumcised had history of offering sexual contact vs. four (2.9%) among circumcised women which constitute a statistically significant difference. Additionally, 14 (10%) of circumcised women reported history of violence against them by their husband while only 2 (3.3%) reported so among the non-circumcised.

**Table 3:** Comparison of sexual function between two groups of circumcised and non-circumcised women

**Variables**		**Non FGM (n=60) n (%)**	**FGM (n=140) n (%)**	***P*-Value**
**Sexual status**	Low desire	16(26.7)	68(48.6)	<0.001
	High desire	23(38.3)	12(8.6)	
	placeCityNormal	21(35.0)	60(42.8)	
**Demand for sexual contact by**	Men	44(73.3)	130(92.9)	<0.001
	Woman	15(25.0)	4(2.9)	
	Both	1(1.7)	6(4.3)	
**Frequency of intercourse per month**	4 ≤	17(28.3)	20(14.3)	0.051
	4–20	40(66.7)	117(83.6)	
	20 ≥	3(5.0)	3(2.1)	
**History of Sexual violence by husband**	No	58(96.7)	126(90.0)	0.111
	Yes	2(3.3)	14(10.0)	

* FGM - Female Genital Mutilation

### *P*-Value based on Chi-square/Fisher’s exact test

There were significant correlation between low quality of life (r=0.42, *P*-value<0.000) and low level of general health(r= −0.65, *P*-value<0.000) with low sexual function trait.

The common type of mutilation in Iran was type I or clitoridectomy and all the FGM participants were circumcised by removing a part or the whole of clitoris. Regarding viewpoints of circumcised women about why they had been under FGM, 64 (45.7%) reported that religious beliefs, traditional rituals, and customs were the main factors of FGM while 58 (41.4%) of participants did not share any understanding of why they have been circumcised. Additionally, 80 (57.1%) of circumcised women reported that religious beliefs and traditional rituals were the main reason for their families and mothers to opt for the FGM procedure for their daughters while 45 (32.9%) reported that cultural beliefs about controlling sexual desire among women were main reasons for mothers to select FGM. According to the women participating in the study, men including fathers had no role on FGM in the studied area. All women were circumcised with a razor; 118 (84.3%) of participants responded that gypsy women were responsible for cutting the girls’ genital organs; 15 (10.7%) responded that relatives did so, and 7 (5%) said that this was done by local women. As regards the age when study participants had been circumcised, 72 (51.4%) of women reported that they were circumcised before age 3 and did not remember this, 65 (46.5%) had been 4–12 yr old, and 3 (2.1%) were older than 12 yr and they could recall this unpleasant event. Additionally, participants reported that they had been subjected to FGM based on their mothers and grandmothers’ request. As for the next generation (the daughters of study participants), none of the respondents reported having subjected their daughters to FGM.

The results of Hotelling’s T-square use for simultaneous comparisons of dimensions between FGM and non-FGM women showed that there were significant differences in QoL and FSFI scores while there was no significant difference in mental well-being score between the two groups (Table 4).

**Table 4:** Simultaneous comparison of dimensions of FSFI,WHOQOL-BREF and GHQ-28 between FGM and non-GGM women

	**Wilks' Lambda Value**	**F**	**df1**	**df2**	***P*-Value**
**GHQ-28**	0.980	0.983b	4.000	195.000	0.418
**WHOQOL-BREF**	0.934	2.720b	5.000	194.000	0.021
**FSFI**	0.732	11.795b	6.000	193.000	<.001

Results based on Multivariate Tests of Hotelling's T-square

## Discussion

The findings of the current study show that women with the history of genital mutilation happen in some parts of Iran similarly to other countries in the Middle East. In particular, it had been common previously between the Arab and Kurds because of cultural and religious beliefs of Sunni Muslims living in the west and south of Iran. However, these practices have dramatically decreased within recent years.

In this study, the proportion of women with FGM was as high as 70% among studied participants in the previous years but it has seen a decreasing trend of FGM among young women in compare of old women and none of the study participants reported FGM on their daughters. Although our study was not conducted on a community level, our findings are comparable with the results of the studies conducted in high prevalence communities such as 27 African countries, Yemen and Iraqi Kurdistan (26–28). The prevalence that we estimated is in line with studies from some ethnic and religious groups located in the west and south of Iran, where most people are Sunni (29).

One of the prominent facets and facts about circumcising of young girls is the exclusive role of women and mothers in conducting circumcision of girls. In other words, men have no determining role with regard to this practice. Similarly, to other nations and countries (3, 5), findings of the current study indicate that women are responsible for FGM in the west of Iran. Fortunately, in the recent years, mothers in Iran are not requesting that their daughters be subjected to circumcision. The rate of FGM is reduced considerably because when it was asked all participants, none of them reported history of circumcision on their daughters but it is needed to systematic research to measure the current rate of FGM among young girls.

Besides ethnic and religious factors, the likelihood of women undergoing FGM might be related to low literacy level among the rural population. In this study, FGM was significantly associated with some variables, including unemployment and lower literacy of parents. Therefore, the practice of FGM is pertinent to certain social and ethnic groups, which is in line with findings in Ethiopia (30) and Egypt. More than 80% of the circumcised women were illiterate and unemployed and that the probability of FGM declined with educational level while being higher among women in lower social strata (31). Low education level and consequent lower level of health knowledge, unemployment and its related poverty in line with living in the deprived rural area might have posed these women at risk of becoming victims of FGM in the studied sample of Kurdish population in Iran.

Clitoris, which is a sexually sensitive tissue, is removed partially or totally in any type of FGM. There is increasing evidence that FGM damages sexual function (3, 14), and findings of the current study demonstrate that FGM was significantly associated with diminished female sexual function. In addition, all domains of female sexual function namely desire, arousal, lubrication, orgasm, satisfaction, and pain could be affected because of FGM; this is consistent with findings of a recent case-control study in Egypt (32). In contrast, in Saudi Arabia was reported no difference in mean desire score or pain score between FGM and non-mutilated females; however, statistically significant differences were reported between the two groups in arousal, lubrication, orgasm, and satisfaction, as well as the overall sexual function score(14). Additionally, as a systematic review and a meta-analysis have concluded, women subjected to FGM significantly more likely reported dyspareunia, the absence of sexual desire, and reduced sexual satisfaction (33).

Additionally, numerous studies have identified depression, anxiety and post-traumatic stress disorder (PTSD) as potential consequences of FGM (34–36). FGM has psychological and mental effects, our study did not reveal discrepancies in mental wellbeing between circumcised and noncircumcised women, and thus, this hypothesis can be further tested in larger scale studies paying more attention to mental health of these women. Despite progressive improvement about health indices and quality of health care services, human society is facing phenomenon of female circumcision, which threatens the health of girls where they become victims of inhumane practices based on certain cultural beliefs. Nowadays, two types of health promotive approaches are needed to overcome FGM phenomenon. First, preventing female circumcision among child girls and secondly, supporting circumcised girls and women who are suffering consequences of FGM.

This study had a number of limitations. First, the cross-sectional study was conducted on a convenience sample of women in Piranshahr County; as a result, our findings cannot be generalized to all Sunni and Kurdish women and other populations of Iran. In addition, the study used self-reported measures that could lead to inaccurate recall and/or reporting. Furthermore, some measurements were conducted with the use of a researcher-designed questionnaire for collecting socio-demographic and health data, rather than using previously standardized instruments, which may have resulted in lack of comparability with other studies, inaccuracies, and misclassification. Nevertheless, this was the first study of this kind among the under-studied population of Kurdish women.

## Conclusion

Many of the Kurdish girls in the county of Piranshahr were circumcised in the past decades, and clitoridectomy was the most common type of mutilation impairing sexual function of mutilated women. Nevertheless, no differences were found between circumcised and non-circumcised women in quality of life and mental wellbeing, which probably confirms that clitoridectomy, is a less harmful procedure in comparison to other types of FGM. Additionally, none of the mutilated women allowed that their daughters be subjected to FGM which indicate a progressive reduction in the rate of female genital mutilation in recent years.

## Ethical considerations

Ethical issues (Including plagiarism, informed consent, misconduct, data fabrication and/or falsification, double publication and/or submission, redundancy, etc.) have been completely observed by the authors.
